# Hampstead General Hospital

**Published:** 1904-05-14

**Authors:** 


### HAMPSTEAD GENERAL HOSPITAL.

Mr. Ernest Collins, the Chairman of the Council of the Hampstead General Hospital, introduced a novel method of raising funds into the proceedings of the Biennial Festival Dinner of that institution which was held at the Grand Hotel, under the presidency of the Lord Mayor. Stigmatising as a disgrace to a wealthy neighbourhood like Hampstead the fact that although they set out to collect £2,000, the total amount announced by the secretary had only reached the sum of £1,810, he appealed to those present not to allow it to be said that they had failed to raise that sum by a paltry £190, and announced that a friend of his, not connected with the neighbourhood, had written to say that if the amount raised did not come up to what was required, he would be willing to give a further £50 towards the deficit provided the deficit was subscribed. "Surely," said Mr. Collins, "we are not going to lose that £50 for want of collecting amongst ourselves another £140." An offer of £50 from one of the guests was promptly received, and then, urged by Mr. Collins's strenuous appeals, two sums of £20 each, one of ten and three of five guineas brought the total up to £165. There, however, the response to the appeal stuck, until at last, seeing that he could get no more out of the company, Mr. Collins said "Well, gentlemen, if you won't subscribe the balance, I will do it myself," and amid cheers those present congratulated themselves that Hampstead had done what was expected of it and had raised the sum of £2,000 to assist the local hospital to carry on its excellent work during the next two years.

Among those present at the dinner were the Mayor of Hampstead (Mr. E. Collingwood Andrews), Alderman and Sheriff Sir John Knill, Bart., Sir S. Maryon-Wilson, Bart., Sir John Glover, Colonel Sir Howard Melliss, K.C.S.I, and Mr. Edward Bond, M.P.

After the usual loyal and other toasts, the Lord Mayor proposed the toast of the evening, "Prosperity to the Hampstead General Hospital," which he said had been established now for a quarter of a century. The hospital started in one small house, and now occupied four houses; but, as his hearers could readily appreciate, the difficulties of successfully carrying on a hospital in four houses were great, and about eighteen months ago it was resolved to build a new institution—one more suitable to a great neighbourhood than the present one. He noticed that in order to provide money for this new building the managers had been obliged to expend the Reserve Fund, a proceeding very much to be deprecated, but one from which in this instance there was no escape. The Hampstead General Hospital, unfortunately, lay out of the beaten track—people of the West End did not see it, and had no opportunities of judging of the wants of the neighbourhood—and so the hospital was thrown back, as it were, upon the immediate neighbourhood for its support. Speaking of the work of the hospital during the past year, the Lord Mayor said there had been 387 in-patients treated, the average number of beds occupied being 25.

Mr. W. F. Malcolm, the treasurer of the hospital, in responding, hoped that the clergy of Hampstead, after arranging for the Hospital Sunday Fund collection this year, would devote a special Sunday to a collection for the Hampstead Hospital. He announced that the contractors for the new Tube Railway from Hampstead (Messrs. Price and Reeves), many of whose men had been treated at the Hospital, had contributed 100 guineas to the Lord Mayor's list. In sending their contribution they wrote as follows:—"We wish to express to you our sincere thanks for the very skilful and careful attention which our accident cases have received." This was a very valuable testimony to the good work done by the hospital, and was an admirable example to all employers whose workpeople are benefited by the institution. He was pleased to tell them that the new hospital was making considerable progress towards completion, but when he remembered the difficulties they had always had in providing funds for working the present hospital, which was something like 50 per cent. smaller than the one they were now erecting, he had to confess that he was filled with anxiety as to where they were to obtain the necessary working expenses in the future. He could only hope that the new hospital would become better known in the neighbourhood. It was singular to find that a neighbourhood like Hampstead, a neighbourhood of wealthy and charitable people, should be unaware of the existence of their local hospital. He could only attribute it to the fact that the hospital was shut out of sight to the great majority of the Hampstead people. Speaking of voluntary compared with rate-supported hospitals, he hoped the change to rate support would never come—certainly that it would never come in his time. Each hospital now, under the voluntary system, was on its best behaviour; each had to do its best work, and faults in management were speedily noted by the Visitors from the great Funds and as speedily set right, or the hospital suffered in pocket. Under the rate-supported system there would be no incentive for a particular hospital to do any better than its neighbours. Under the voluntary system the hospitals had the unpaid assistance of a great number of devoted medical men who gave their valuable time to the work of the hospitals. All that would be changed under a system of municipal hospitals. Furthermore he believed that municipal hospitals for London would mean an increase in the rates of 3d. in the £. That in Hampstead would mean a contribution each year of about £12,000 towards the hospitals of London, as Hampstead, a wealthy neighbourhood, would have to assist the poorer neighbourhoods. He was sure that the managers of the Hampstead Hospital would be quite willing to compound with the inhabitants of Hampstead for the equivalent of a 1d. rate, or £4,000 a year, and if they could be assured of that sum yearly they would have all that they required.

"The Council and the Medical Staff," proposed by Mr. Henry Stedall, and responded to by Mr. Ernest Collins (the Chairman of the Council) and Dr. W. Heath Strange (one of the founders of the hospital), concluded the toast list.

